# End-stage renal disease and outcome in a surgical intensive care unit

**DOI:** 10.1186/cc13167

**Published:** 2013-12-23

**Authors:** Mareike Apel, Vivian PL Maia, Mohamed Zeidan, Claudia Schinkoethe, Gunter Wolf, Konrad Reinhart, Yasser Sakr

**Affiliations:** 1 Department of Anesthesiology and Intensive Care, Friedrich-Schiller-University Hospital, Erlanger Allee 103, 07743 Jena, Germany; 2 Department of Anesthesiology and Intensive Care, Theodor Bilharz Institute, El-Nile St., Warrak El-Hadar, 12411 Embaba, Giza, Egypt; 3 Department of Internal Medicine III, Friedrich-Schiller-University Hospital Erlanger Allee 103, 07743 Jena, Germany

## Abstract

**Introduction:**

End-stage renal disease (ESRD) is associated with an increased propensity for critical illness, but whether ESRD is independently associated with a greater risk of death after major surgical procedures is unclear.

**Methods:**

This was a retrospective analysis of prospectively collected data from all adult (>18 years) patients admitted to a 50-bed surgical intensive care unit (ICU) between January 2004 and January 2009. ESRD was defined as the need for chronic peritoneal dialysis or hemodialysis for at least 6 weeks prior to ICU admission. We used multivariable logistic regression analysis and propensity-score matching to adjust for possible confounders.

**Results:**

In total, 12,938 adult patients were admitted during the study period; 199 patients had ESRD at ICU admission, giving a prevalence of 1.5%. Patients with ESRD were more likely to be male (72.9% versus 63.0%, *P* = 0.004) and had higher severity scores, a higher incidence of diabetes mellitus and cirrhosis, and a lower incidence of cancer at ICU admission than those without ESRD. Patients with ESRD were more likely to have any type of organ failure at ICU admission and during the ICU stay. Patients with ESRD had higher ICU and hospital mortality rates (23.1% and 31.2% versus 5.5% and 10.0%, respectively, *P* <0.001 pairwise) and longer ICU length of stay (2 (1 to 7) versus 1 (1 to 3) days, *P* <0.001). In multivariable logistic regression analysis, ESRD was independently associated with a greater risk of in-hospital death (odds ratio = 3.84, 95% confidence interval 2.68 to 5.5, *P* <0.001). In 199 pairs of patients, hematologic and hepatic failures were more prevalent, ICU and hospital mortality rates were higher (23.1% versus 15.1% and 31.2% versus 19.1%, *P* <0.05 pairwise), and ICU length of stay was longer (2 (1 to 7) versus 1 (1 to 7) days, *P* <0.001) in patients with ESRD.

**Conclusions:**

In this large cohort of surgical ICU patients, presence of ESRD at ICU admission was associated with greater morbidity and mortality and independently associated with a greater risk of in-hospital death. Our data can be useful in preoperative risk stratification.

## Introduction

The prevalence of chronic kidney disease is increasing worldwide [1,2]. The progressive nature of this chronic health problem and the ensuing end-stage renal disease (ESRD) creates a considerable burden on global health-care resources [3]. The annual incidence of ESRD has doubled over the past decade to reach about 100 to 336 new patients per million population [3,4]. Patients with ESRD have a high propensity for critical illness and require intensive care unit (ICU) admission 25 times more frequently than patients without ESRD [5,6]. The prevalence of ERSD in ICU patients ranges between 1.3% and 7.3% and its presence is associated with a higher degree of morbidity and mortality in these patients [5,7,8].

Whether ESRD is associated per se with a higher risk in critically ill patients, independent of the severity and nature of the critical illness, remains unclear. In a large cohort of patients admitted to 170 adult ICU patients in England, Wales, and Northern Ireland, ESRD was associated with a higher risk of in-hospital death after adjusting for possible confounders [7]. This result was not, however, confirmed in a large database of ESRD patients admitted to 11 Canadian ICUs [5]. However, these studies [5,7] included mixed medical and surgical ICU patients, with a high proportion of medical admissions. Data on the possible impact of ESRD on outcome after major surgical procedures are scarce. Such information may be useful in preoperative risk stratification of surgical patients and hence could improve clinical decision making in these patients. It may also be interesting to identify the patterns of non-renal organ dysfunction/failure and predictors of poor outcome in ESRD patients admitted to the ICU after major surgical procedures. The aims of our study were, therefore, to test the hypothesis that ESRD is independently associated with a higher risk of death after major surgical procedures and to identify possible risk factors for in-hospital death in these patients.

## Materials and methods

The study was approved by the institutional review board of Friedrich Schiller University Hospital (Jena, Germany). Informed consent was waived because of the retrospective, anonymous nature of the analysis. We included all adult (>18 years old) patients admitted to our 50-bed surgical ICU from January 2004 to January 2009. For patients admitted to the ICU more than once, only the first admission was considered.

### Data collection

Data were collected from vital sign monitors, ventilators, and infusion pumps and automatically recorded by a patient data management system (Copra System GmbH, Sasbachwalden, Germany). This system provides staff with complete electronic documentation, order entry (for example, medications), and direct access to laboratory results. Documentation in our ICU is exclusively electronic. Data recorded prospectively on admission include age, gender, serum parameters, primary and secondary admission diagnoses, and surgical procedures. Primary and secondary diagnoses are recorded by using codes from the International Classification of Diseases-10.

The Simplified Acute Physiology Score II (SAPS II) [9] was calculated on admission, and the Sequential Organ Failure Assessment (SOFA) score [10] was calculated daily by the physician in charge of the patient by using a special sheet. A plausibility check of the automatically transmitted data was performed by the attending physician before calculating the final scores. In sedated patients, the Glasgow Coma Scale prior to initiation of sedation was considered. Hospital mortality and hospital discharge dates were available for all patients from the electronic hospital records.

### Definitions

ESRD was defined as the need for chronic peritoneal dialysis or hemodialysis for at least 6 weeks prior to the time of admission to the ICU. In patients with ESRD following acute renal failure, 3 months were required to establish the diagnosis of ESRD. Diabetes mellitus refers to types 1 and 2 diabetes mellitus. SOFA_max_ was defined as the maximum SOFA score recorded during the ICU stay, and SOFA mean as the mean value during the ICU stay [10].

### Statistical analysis

Data were analyzed by using SPSS 13.0 for Windows (SPSS Inc., Chicago, IL, USA) and SAS version 9.1.3 software (SAS Institute Inc., Cary, NC, USA). The Kolmogorov-Smirnov test was used to verify the normality of distribution of continuous variables. Non-parametric tests of comparison were used for variables evaluated as not being normally distributed. Difference testing between groups was performed by using a Wilcoxon test, Mann-Whitney *U* test, chi-square test, and Fisher’s exact test as appropriate.

To define the possible factors associated with poor outcome in the whole cohort we performed a multivariable logistic regression analysis, with in-hospital death as the dependent variable. The variables considered for this analysis were age, sex, SAPS II, SOFA subscores, type of surgery, emergency admissions, and ESRD. Colinearity between variables was excluded before modeling (R^2^ >0.6), and none of the covariates was colinear. A Hosmer and Lemeshow goodness-of-fit test was performed, and odds ratios (ORs) with 95% confidence intervals (CIs) were computed.

To identify the possible risk factors associated with in-hospital death in patients with ESRD, we performed a forward stepwise multivariable logistic regression analysis with in-hospital death as the dependent variable. The variables considered for this analysis were age, sex, SAPS II, SOFA subscores, type of surgery, emergency admissions, the etiology of ESRD, residual urine output (≤500 mL/day and >500 mL/day), and the type of access for dialysis (arterio-venous shunt, central venous catheter, and peritoneal catheter). Covariates were entered in the model (*P* <0.2 on a univariate basis) step-by-step and retained in the model if the *P* value was less than 0.2 after multivariable adjustment.

Propensity scores [11] were obtained through logistic regression of patient characteristics on ESRD status (Additional file 1: Table S1). The propensity score was calculated as the probability based on the final model. A greedy matching technique was used to match individual ESRD patients with those who did not have ESRD, based on propensity scores. The best-matched propensity score was five digits long. Once a match was made, the control patient was removed from the pool. This process was then repeated by using four-digit matching, then three-digit matching, and so on. The process proceeded sequentially to a single-digit match on propensity score. Differences between matched pairs were tested by using a paired *t* test, Wilcoxon test, and McNemar X2 statistics, as appropriate.

All statistics were two-tailed. A *P* value of less than 0.05 was considered to be significant. Continuous variables are presented as mean ± standard deviation or median (25% to 75% interquartile range) and categorical variables as number and percentage, unless otherwise indicated.

## Results

### Characteristics of the study cohort

During the study period, 12,938 patients were admitted to our surgical ICU (mean age = 62.5 years (standard deviation 14.9), male = 63.2%). The characteristics of the study groups on admission to the ICU are shown in Table 1. The prevalence of ESRD on admission to the ICU was 1.5% (n = 199). Diabetic (40.2%) and hypertensive (23.6%) nephropathies were the most common causes of ESRD, followed by inflammatory conditions (chronic glomerulonephritis, interstitial nephritis, chronic pyelonephritis, and nephritic syndrome: 16.5%), congenital diseases (renal hypoplasia and polycystic kidney: 6.5%), and neoplasm (4.0%). Among patients with ESRD, 117 (58.8%) were anuric, whereas 82 patients (41.2%) had residual diuresis prior to ICU admission (≤ or >500 mL/day in 40 and 42 patients, respectively). The predominant mode of dialysis in patients with ESRD was hemodialysis (98.0%); in 64.3% of cases, this was performed through an arterio-venous shunt and in 34% through a right atrial catheter. Only four patients (2%) were treated by peritoneal dialysis.

**Table 1:** Characteristics of the study group on admission to the intensive care unit according to the presence of end-stage renal disease

	**All patients**	**ESRD**	**No ESRD**	***P *** **value**
	**(n = 12,938)**	**(n = 199)**	**(n = 12,739)**	
Age in years, mean ± SD	62.5 ± 14.9	62.4 ± 13.1	62.5 ± 14.9	0.509
Male, n (%)	8,176 (63.2)	145 (72.9)	8,031 (63.0)	0.004
Severity scores, mean ± SD				
SAPS II	38.3 ± 18.6	56.3 ± 20.5	38.0 ± 18.4	<0.001
SOFA	6.0 ± 3.9	10.6 ± 4.2	6.0 ± 3.8	<0.001
SOFA (without renal points)	5.7 ± 3.6	7.5 ± 4.2	5.6 ± 3.6	<0.001
Type of surgery, n (%)				
Cardiothoracic	5,409 (41.8)	84 (42.2)	5,325 (41.8)	0.145
Gastrointestinal	2,780 (21.5)	59 (29.6)	2,721 (21.4)	<0.001
Neurosurgery	2,030 (15.7)	8 (4.0)	2,022 (15.9)	<0.001
Trauma	523 (4.0)	9 (4.5)	514 (4.0)	0.229
Other surgery^a^	2,196 (16.9)	39 (19.6)	2,157 (16.9)	0.001
Comorbidities, n (%)				
Diabetes mellitus	6,749 (52.2)	148 (74.4)	6,601 (51.8)	<0.001
Arterial hypertension	2,785 (21.5)	47 (23.6)	2,738 (21.5)	0.469
Cancer	2,501 (19.3)	19 (9.5)	2,482 (19.5)	<0.001
Heart failure	1,645 (12.7)	33 (16.6)	1,612 (12.7)	0.099
Cirrhosis	472 (3.6)	19 (9.5)	453 (3.6)	<0.001

^a^Renal/urinary tract, obstetric/gynecologic, ear, nose, and throat, and maxillofacial surgeries. ESRD, end-stage renal disease; SAPS II, Simplified Acute Physiology Score II; SD, standard deviation; SOFA, Sequential Organ Failure Assessment.

Patients with ESRD were more likely to be male (72.9% vs. 63.0%, *P* = 0.004), had higher severity scores, and had a greater incidence of diabetes mellitus and cirrhosis and lower incidence of cancer on admission to the ICU than those without ESRD (Table 1). Gastrointestinal surgery was more common (29.6% vs. 21.4%, *P* <0.001) and neurosurgical procedures less common (15.9% vs. 4.0%, *P* <0.001) in patients with ESRD compared with those without ESRD. Serum urea, serum creatinine, leukocyte count, and serum potassium were higher whereas hematocrit values, platelet counts, pH, serum bicarbonate, and serum sodium levels were lower in patients with ESRD than those without ESRD (Additional file 1: Table S2).

### Morbidity and mortality

The most prevalent organ failures on admission to the ICU and at any time during the ICU stay were cardiovascular (46.2% and 49.5%) followed by central nervous system (36.7% and 42.4%) and respiratory (24.1% and 30.9%) failures. Patients with ESRD were more likely to have any type of organ failure on admission to the ICU and at any time during the ICU stay than those without ESRD (Table 2). Notably, hematologic failure occurred up to 4 times more often in patients with ESRD (21.6% vs. 4.5%, *P* <0.001) than in other patients. Consequently, SOFA_max_ with (11.5 ± 4.6 vs. 6.4 ± 4.2, *P* <0.001) or without (7.5 ± 4.2 vs. 5.6 ± 3.6, *P* <0.001) renal points was higher in patients with ESRD than in those without ESRD.

**Table 2:** Morbidity and mortality of the study group according to the presence of end-stage renal disease

	**All patients**	**ESRD**	**No ESRD**	***P *** **value**
	**(n = 12,938)**	**(n = 199)**	**(n = 12,739)**	
Organ failure, n (%)				
- On admission to the ICU				
Cardiovascular	5,974 (46.2)	135 (67.8)	5,839 (45.8)	<0.001
CNS	4,747 (36.7)	90 (45.2)	4,657 (36.6)	0.012
Respiratory	3,120 (24.1)	68 (34.2)	3,052 (24.0)	0.001
Renal	390 (3.0)	199 (100)	237 (1.9)	<0.001
Hematologic	309 (2.4)	27 (13.6)	282 (2.2)	<0.001
Hepatic	215 (1.7)	15 (7.5)	200 (1.6)	<0.001
- At any time during ICU stay				
Cardiovascular	6,407 (49.5)	144 (72.4)	6,263 (49.2)	<0.001
CNS	5,489 (42.4)	112 (56.3)	5,377 (42.2)	<0.001
Respiratory	3,992 (30.9)	88 (44.2)	3,904 (30.6)	<0.001
Renal	630 (4.9)	199 (100)	431 (3.4)	<0.001
Hematologic	618 (4.8)	43 (21.6)	575 (4.5)	<0.001
Hepatic	405 (3.1)	24 (12.1)	381 (3.0)	<0.001
SOFA_max_ scores, mean ± SD				
SOFA_max_	6.5 ± 4.3	11.5 ± 4.6	6.4 ± 4.2	<0.001
SOFA_max_ without renal points	5.7 ± 3.6	7.5 ± 4.2	5.6 ± 3.6	<0.001
ICU mortality, n (%)	746 (5.8)	46 (23.1)	700 (5.5)	<0.001
Hospital mortality, n (%)	1,330 (10.3)	62 (31.2)	1,268 (10.0)	<0.001
ICU LOS in days, median (IQR)	1 (1–3)	2 (1–7)	1 (1–3)	<0.001

CNS, central nervous system; ESRD, end-stage renal disease; ICU, intensive care unit; IQR, interquartile range; LOS, length of stay; SD, standard deviation; SOFA, Sequential Organ Failure Assessment; SOFA_max_, maximum Sequential Organ Failure Assessment score recorded during the intensive care unit stay.

The overall ICU and hospital mortality rates were 5.8% and 10.3%, respectively, and the median ICU length of stay (LOS) was 1 day (interquartile range: 1–3 day). Patients with ESRD had higher ICU and hospital mortality rates (23.1% and 31.2% vs. 5.5% and 10%, respectively, *P* <0.001 pairwise) and longer ICU LOS (2 (1–7) vs. 1 (1–3) days, *P* <0.001) than those without ESRD. The most common causes of death in the ICU were sepsis-related multiorgan failure (52.2%), bleeding complications (15.2%), cardiogenic shock (13.0%), and electrolyte disturbances (10.9%).

### Multivariable analysis and propensity score matching

In multivariable logistic regression analysis with hospital mortality as the dependent variable, ESRD was independently associated with a greater risk of in-hospital death (OR = 3.84, 95% CI: 2.68-5.5, *P* <0.001) after adjustment for age, gender, comorbidities, SAPS II, type of surgery, and SOFA subscores on admission to the ICU (Additional file 1: Table S3).

In 199 pairs of patients matched according to a propensity score, age, sex, comorbid conditions, severity scores, and non-renal organ failure on admission to the ICU were similar between patients with and those without ESRD (Table 3). The type of surgery prior to admission to the ICU was similar between the matched groups apart from a lower prevalence of neurosurgical procedures in patients with ESRD than in those who did not have ESRD (4.0% vs. 14.6%, *P* <0.001). Although the prevalence of respiratory, cardiovascular, and central nervous system organ failures was similar between the matched groups throughout the ICU stay, hematologic (21.6% vs. 11.6%, *P* = 0.015) and hepatic (12.1% vs. 8.5%, *P* = 0.03) organ failures were more prevalent in patients with ESRD than in their propensity score-matched pairs (Table 4). ICU and hospital mortality rates were higher (23.1% vs. 15.1% and 31.2% vs 19.1%, respectively, *P* <0.05 pairwise) and ICU LOS was longer (2 (1–7) vs. 1 (1–7) days, *P* <0.001) in patients with ESRD compared with their propensity score-matched pairs.

**Table 3:** Characteristics of the propensity score-matched groups on admission to the intensive care unit

	**ESRD**	**No ESRD**	***P *** **value**
	**(n = 199)**	**(n = 199)**	
Age in years, mean ± SD	62.4 ± 13.1	61.4 ± 14.4	0.524
Male, n (%)	145 (72.9)	161 (80.9)	0.057
Severity scores, mean ± SD			
SAPS II	41.95 ± 19.35	40.39 ± 17.22	0.422
SOFA^a^	7.47 ± 4.20	7.04 ± 3.52	0.458
Comorbidities, n (%)			
Diabetes mellitus	148 (74.4)	141 (70.9)	0.431
Arterial hypertension	47 (23.6)	33 (16.6)	0.080
Heart failure	33 (16.6)	21 (10.6)	0.079
Cirrhosis	19 (9.5)	20 (10.1)	0.866
Cancer	19 (9.5)	23 (11.6)	0.514
Type of surgery on admission day, n (%)			
Cardiothoracic	84 (42.2)	83 (41.7)	0.919
Digestive	59 (29.6)	53 (26.6)	0.504
Neurosurgery	8 (4.0)	29 (14.6)	<0.001
Trauma	9 (4.5)	8 (4.0)	0.804
Others	39 (19.6)	26 (13.1)	0.122
Organ failure, n (%)			
Cardiovascular	135 (67.8)	126 (63.3)	0.342
CNS	90 (45.2)	76 (38.2)	0.155
Respiratory	68 (34.2)	55 (27.6)	0.158
Hematologic	27 (13.6)	15 (7.5)	0.050
Hepatic	15 (7.5)	9 (4.5)	0.206

^a^Without renal points. CNS, central nervous system; ESRD, end-stage renal disease; SAPS II, Simplified Acute Physiology Score II; SD, standard deviation; SOFA, Sequential Organ Failure Assessment.

**Table 4:** Sequential Organ Failure Assessment scores and organ failure during the intensive care unit stay, and mortality rates in the propensity score-matched subgroups

	**ESRD**	**No ESRD**	***P *** **value**
	**(n = 199)**	**(n = 199)**	
SOFA_max_ scores, mean ± SD			
SOFA_max_ (with renal points)	11.45 ± 4.62	8.31 ± 4.41	<0.001
SOFA_max_ (without renal points)	8.61 ± 4.96	7.79 ± 4.13	0.166
Organ failure, n (%)			
Renal	199 (100.0)	7 (3.5)	<0.001
Cardiovascular	144 (72.4)	134 (67.3)	0.516
CNS	112 (56.3)	100 (50.3)	0.144
Respiratory	88 (44.2)	79 (39.7)	0.658
Hematologic	43 (21.6)	23 (11.6)	0.015
Hepatic	24 (12.1)	17 (8.5)	0.030
ICU LOS in days, median (IQR)	2 (1–7)	1 (1–7)	<0.001
Hospital mortality, n (%)	62 (31.2)	38 (19.1)	0.006
ICU mortality, n (%)	46 (23.1)	30 (15.1)	0.041

CNS, central nervous system; ESRD, end-stage renal disease; ICU, intensive care unit; IQR, interquartile range; LOS, length of stay; SD, standard deviation; SOFA, Sequential Organ Failure Assessment; SOFA_max_, maximum Sequential Organ Failure Assessment score recorded during the intensive care unit stay.

### Predictors of poor outcome in patients with ESRD

In a multivariable analysis in patients with ESRD (Additional file 1: Table S4), the use of central venous catheters for dialysis (OR: 3.30, 95% CI: 1.36-8.04, *P* = 0.009) and higher hepatic SOFA subscores (OR = 1.60, 95% CI: 1.07-2.4, *P* = 0.022) were independently associated with an increased risk of in-hospital death. Female sex, admission after cardiac versus non-cardiac procedures, and residual urinary output of greater than 500 mL versus anuria were independently associated with a lower risk of in-hospital death (Additional file 1: Table S4).

## Discussion

The main findings of our study were that (1) ESRD was associated with a high incidence of comorbidities and high degree of severity of illness on admission to the ICU; (2) patients with ESRD were more likely to have any type of organ failure during the ICU stay than those without ESRD, especially hematologic failure; (3) ESRD was independently associated with a greater risk of in-hospital death in multivariable analysis; (4) mortality rates were higher, ICU LOS was longer, and hematologic and hepatic organ failures were more prevalent in patients with ESRD compared with propensity score-matched pairs; and (5) the use of central venous vascular access and higher hepatic SOFA subscores were independently associated with increased risk of in-hospital death, whereas female sex, admission after cardiac procedures, and residual urinary output of greater than 500 mL versus anuria were independently associated with decreased risk of in-hospital death in patients with ESRD.

The prevalence of ESRD on admission to the ICU in our study was 1.5%, which lies within the lower spectrum of the prevalence rates reported in previous studies that included mostly medical ICU patients [5,7,8]. Indeed, the presence of ESRD may have influenced the decision to perform surgical procedures in our hospital, especially those performed on an elective basis. For this reason, in addition to the single-center nature of the study, we cannot extrapolate these data to other cohorts.

As expected, we found that ESRD was associated with a high incidence of comorbidities on admission to the ICU. This can be explained by the contribution of these comorbidities to the pathophysiology of ESRD, especially diabetes mellitus, which is the most common cause of ESRD in developed countries [3]. The higher prevalence of liver cirrhosis in patients with ESRD compared with others may be attributed to the high incidence of hepatitis C and D in these patients [11]. Male sex is also well recognized as a risk factor for chronic kidney disease [3,12,13], which explains the predominance of men (72.9%) among the patients with ESRD in our study. In addition, the most frequent underlying diseases were diabetes and hypertension, both of which suggest at least an alteration of vascular control. This association with a vascular injury related to ESRD might have contributed to the worth outcome in these patients.

In our study, organ failure occurred more frequently in patients with ESRD than in those without ESRD. Impaired renal function is a major risk factor for cardiovascular morbidity and mortality [14]. Several factors may contribute to the increased incidence of cardio-respiratory dysfunction in patients with ESRD, including left ventricular hypertrophy, rapid electrolyte shifts during dialysis, QT dispersion, sympathetic over-activity, and deposition of calcium-phosphate precipitants within arteries [14,15]. Acute pulmonary infections, perioperative volume overload, excessive inter-dialytic weight gain, and primary cardiac events may also contribute to respiratory failure in patients with ESRD. The higher prevalence of hepatic failure in our patients may be explained by the higher incidence of liver cirrhosis in these patients compared with those without ESRD. The impairment of renal elimination of hypnotic and sedative agents given perioperatively in patients with ESRD [14] may explain the high occurrence of neurological failure in these patients. Interestingly, we found that hematologic failure occurred up to 4 times more frequently in patients with ESRD than in those without. Patients on chronic dialysis have abnormalities in the cellular and plasma systems regulating blood homeostasis, which may be caused by uremic plasma associated with exposure of blood to the hemodialysis membranes and tubing causing increased thrombotic and bleeding risks [16]. Platelet dysfunction is a common occurrence in patients with ESRD, as a result of intrinsic platelet abnormalities and impaired platelet-vessel wall interaction, and is probably responsible for the hemorrhagic tendencies in these patients [16]. Recently, Darlington and colleagues [17] reported that 42.9% of patients with ESRD had a functional hypocoagulable state, using thromboelastography, compared with 8.9% in the control group.

In our study, ESRD was independently associated with a greater risk of in-hospital death after adjusting for possible confounders. We were able to confirm this finding by using two statistical techniques, namely, logistic regression multivariable analysis and propensity score matching. To the best of our knowledge, our study is the first to investigate this issue in a purely surgical cohort of ICU patients. Our data support the notion that ESRD per se may contribute, independently of other comorbidities and severity of illness, to the risk of in-hospital death after major surgical procedures. Several small studies [8,18-21] and two large multicenter studies [5,7] have reported contradictory results, with some confirming [6-8,18] and others [5,19-21] disputing the independent association between ESRD and poor outcome from critical illness. The discrepancy in the results of these studies may be explained by differences in case mix and local practices. Indeed, these studies included mixed populations of medical and surgical ICU patients (65% to 73% non-surgical). Nevertheless, our results are in agreement with the results of a large multicenter study that included 270,972 adult ICU patients admitted to 170 ICUs in the UK (56% non-surgical cases) and that reported that ESRD was associated with a greater risk of in-hospital death after adjusting for baseline characteristics, severity of illness, and comorbidities [7]. Interestingly, these authors [7] reported that the ESRD population had higher mortality rates with lower numbers of non-renal organ failures, confirming the independent influence of ESRD on the risk of death in these patients.

The poor outcome in patients with ESRD is probably multifactorial. These patients likely lack physiological reserve, as evident from the high severity of illness and the associated comorbid conditions on admission to the ICU. Patients with ESRD may also have disturbed immunological responses, favoring infectious complications [7]. Perioperative patient management, especially volume administration, may also have contributed to the unfavorable outcome. The need for vascular access to provide hemodialysis in the ICU postoperatively and the frequent occurrence of electrolyte disturbances in patients with ERDS are other risk factors that may have influenced outcomes. The results of the propensity score matching in our study provide more insight about the possible mechanisms by which ESRD may influence outcome in patients with ESRD. Although the 199 pairs were well matched according to baseline characteristics, severity of illness, type of surgery, and the frequency of individual non-renal organ failures on admission to the ICU, patients with ESRD had a higher prevalence of hematologic and hepatic failures in the ICU. Hematologic failure occurred almost twice as often in patients with ESRD as in their propensity score-matched pairs. As discussed above, hematologic disturbances in patients with ESRD may be associated with an increased risk of both bleeding and thrombosis [16], which may contribute to the pathogenesis of multiorgan failure and poor outcome. As we used SOFA subscores to assess organ dysfunction/failure, hematologic failure in our study refers to thrombocytopenia. In a prospective cohort of 329 critically ill patients, Vanderschueren and colleagues [22] found that thrombocytopenia was a risk marker for mortality, independent of and complementary to established severity of disease indices, and that both a low nadir platelet count and a large decrease in platelet count predicted a poor outcome in these patients.

Several studies have reported risk factors associated with poor outcome in patients with ESRD in various settings, including age [5,7,23], higher severity scores [5-7], associated malignancy [5], the presence of infection [5-7,24], the need for mechanical ventilation [5,8], and anemia [25,26]. Data on risk factors for death in ESRD patients undergoing major surgical procedures are scanty. But this information may be important in risk stratification of patients prior to major surgical procedures. Previous studies have reported that the extent of organ dysfunction failure is associated with the risk of death in ICU patients with ESRD [5,7]. In our study, we identified hepatic SOFA subscores as being independently associated with an increased risk of in-hospital death. The use of central venous catheters for dialysis was also independently associated with an increased risk of in-hospital death. This observation confirms the results of a Canadian cohort of 578 ICU patients with ESRD [6]. This association may have been subject to a bias-by-indication in which sicker patients may have been preferentially dialyzed using central venous access in the ICU; nevertheless, central venous catheters may be associated with additional risks related to catheter placement and risk of infection in the ICU, which may explain, at least in part, the possible association of central venous access and mortality in these patients.

In agreement with the results of a previous study by Shah and colleagues [23], we found that female sex was associated with a more favorable outcome in patients with ESRD than male sex. Sexual dimorphism in the immune response has been correlated to differences in sex steroid hormone concentrations [27-29]. These differences in hormonal secretion may explain the improved survival of critically ill women. We also found that residual urinary output of greater than 500 mL was associated with a decreased risk of in-hospital death in patients with ESRD. This observation may be explained by the ability of kidney in these patients to reduce volume overload, which is known to be associated with deleterious effects on outcome of renal failure in the ICU [30].

Our study has some limitations. First, our analysis is retrospective in nature and our results are only hypothesis-generating. A larger prospective observational trial in surgical ICU patients or in specific subpopulations is warranted to clarify this issue. Second, the multivariable analysis does not take into account unmeasured variables and cannot establish a cause-effect relation. The confounding effect of unmeasured variables, such as perioperative volume management, cannot be excluded. Nevertheless, many relevant variables were considered in our analysis. Third, we included a heterogeneous case mix of surgical ICU patients and were not able to perform subgroup analysis for the different surgical disciplines, because of the relatively small number of patients with ERSD in the subgroups. Finally, the results of our study cannot be extrapolated to populations with other case mixes, such as medical patients. The singe-center nature of the study may be another limiting factor that hinders the extrapolation of data to other cohorts of surgical ICU patients with a different case mix. This should be explored in large-scale multicenter studies. Nonetheless, the absence of major variability in clinical practice in single-center studies, such as ours, may reduce the impact of the possible confounding effect of variability in ICU and hospital organizational issues that may be difficult to adjust for in large-scale multicenter studies.

## Conclusions

In this single-center, large cohort of patients in the surgical ICU, ESRD was associated with higher morbidity and mortality. The presence of ESRD was independently associated with a higher risk of in-hospital death. Our data can be useful in preoperative risk stratification. Further large multicenter studies are warranted to confirm whether these data can be generalized to a wide range of patients in the surgical ICU.

## Key messages

•Postoperative patients with end-stage renal disease (ESRD) have a high incidence of comorbidities and a high degree of severity of illness on admission to the intensive care unit (ICU).

•These patients are more likely to have any type of organ failure during the ICU stay than those without ESRD, especially hematologic failure.

•ESRD was independently associated with a greater risk of in-hospital death.

•The use of central venous vascular access and higher hepatic Sequential Organ Failure Assessment (SOFA) subscores were independently associated with increased risk of in-hospital death, whereas female sex, admission after cardiac procedures, and residual urinary output of more than 500 mL versus anuria were independently associated with decreased risk of in-hospital death in patients with ESRD.

## Abbreviations

CI: confidence interval; ESRD: end-stage renal disease; ICU: intensive care unit; LOS: length of stay; OR: odds ratio; SAPS II: Simplified Acute Physiology Score II; SOFA: Sequential Organ Failure Assessment; SOFAmax: maximum Sequential Organ Failure Assessment score recorded during the intensive care unit stay.

## Competing interests

The authors declare that they do not have any competing interests in relation to the subject of this manuscript.

## Authors’ contributions

MA contributed to the study design and helped collect data and revise the medical charts. YS contributed to the study design and helped perform the statistical analysis and draft the manuscript. KR and GW contributed to the study design and revised the manuscript. VPLM helped collect data, revise the medical charts, perform the statistical analysis, and draft the manuscript. CS helped collect data, revise the medical charts, and draft the manuscript. MZ helped collect data and revise the medical charts. All authors read and approved the final manuscript.

## Supplementary Material

Additional file 1: Table S1 The multivariable model used to obtain the propensity score. **Table S2**. Physiological, acid–base parameters, and serum electrolytes according to the presence of end-stage renal disease (ESRD). **Table S3**. Logistic regression analysis with hospital mortality as the dependent variable in the whole population. **Table S4**. Summary of a multivariable forward stepwise logistic regression analysis with in-hospital death as the dependent variable. Click here for file
