# Supplementary material for: End-stage renal disease and outcome in a surgical intensive care unit
Source: Crit Care. 2013 Dec 23;17(6):R298. doi: 10.1186/cc13167 (PMC4057028; doi:10.1186/cc13167)
Supplement: Additional file 1: Table S1 — The multivariable model used to obtain the propensity score. Table S2. Physiological, acid–base parameters, and serum electrolytes according to the presence of end-stage renal disease (ESRD). Table S3. Logistic regression analysis with hospital mortality as the dependent variable in the whole population. Table S4. Summary of a multivariable forward stepwise logistic regression analysis with in-hospital death as the dependent variable. [file cc13167-S1.doc]

**End-Stage Renal Disease and Outcome in a Surgical Intensive Care Unit**

Mareike Apel MD, Vivian PL Maia MD, Mohamed Zeidan MD PhD, Claudia Schinkoethe MD, Gunter Wolf MD, Konrad Reinhart MD, and Yasser Sakr MD PhD

SUPPLEMENTARY DIGITAL CONTENT

Table 1. The multivariable model used to obtain the propensity score

|  | **B** | **S.E.** | **Wald** | **OR (95% CI)** | **p-value** |
| --- | --- | --- | --- | --- | --- |
| Age | -0.02 | 0.01 | 12.12 | 0.98 (0.97-0.99) | <0.001 |
| Sex | -0.37 | 0.16 | 5.23 | 0.69 (0.50-0.95) | 0.022 |
| Severity scores |  |  |  |  |  |
| SAPS II without kidney | 0.02 | 0.01 | 5.8 | 1.02 (1.00-1.04) | 0.016 |
| SOFA subscores |  |  |  |  |  |
| Respiration | -0.14 | 0.07 | 4.32 | 0.87 (0.78-0.99) | 0.038 |
| Coagulation | 0.41 | 0.08 | 24.92 | 1.50 (1.28-1.76) | <0.001 |
| Liver | 0.04 | 0.09 | 0.17 | 1.04 (0.86-1.25) | 0.683 |
| Cardiovascular | 0.27 | 0.07 | 16.92 | 1.31 (1.15-1.49) | <0.001 |
| GCS | -0.12 | 0.07 | 2.96 | 0.88 (0.77-1.02) | 0.085 |
| Comorbidities |  |  |  |  |  |
| Diabetes mellitus | 1.15 | 0.18 | 40.88 | 3.12 (2.22-4.51) | <0.001 |
| Arterial hypertension | 0.13 | 0.19 | 0.52 | 1.14 (0.80-1.65) | 0.470 |
| Cirrhosis | 0.35 | 0.29 | 1.41 | 1.42 (0.80-2.53) | 0.235 |
| Cancer | -0.66 | 0.25 | 6.86 | 0.52 (0.32-0.85) | 0.009 |
| Heart failure | 0.1 | 0.21 | 0.25 | 1.11 (0.74-1.66) | 0.621 |
| Emergency surgery | 0.01 | 0.18 | 0.001 | 1.01 (0.71-1.42) | 0.975 |
| Constant | -4.75 | 0.36 | 171.1 | 0.01 | <0.001 |

B: coefficient; S.E: standard deviation; OR: odds ratio; CI: confidence interval; SAPS II: Simplified Acute Physiology Score; SOFA: Sequential Organ Failure Assessment; GCS: Glasgow Coma Scale.

Table 2. physiological, acid-base parameters, and serum electrolytes according to the presence of end-stage renal disease (ESRD).

|  | **All patients**  **(n=12938)** | **ESRD**  **(n=199)** | **No ESRD**  **(n=12739)** | **p-value** |
| --- | --- | --- | --- | --- |
| Physiological parameters |  |  |  |  |
| Serum urea, mmol/L | 5.6 (4.3-8.1) | 16.2 (12.0-23.2) | 5.6 (4.3-7.9) | < 0.001 |
| Serum creatinine, µmol/L | 87 (73-110) | 477 (334-624) | 87 (73-108) | < 0.001 |
| Bilirubin, µmol/L | 15 (10-23) | 15 (10-27) | 15 (10-22) | 0.132 |
| Hematocrit, % | 28 (25-33) | 26 (33-29) | 29 (25-33) | < 0.001 |
| Leukocytes, /µL | 12.1 (9.3-16.0) | 13.6 (9.8-17.7) | 12.1 (9.3-16.0) | 0.004 |
| Platelet count, 10³/µl | 160 (118-213) | 126 (77-175) | 161 (119-214) | < 0.001 |
| Acid-base parameters |  |  |  |  |
| pH | 7.30 (7.30-7.30) | 7.20 (7.20-7.30) | 7.30 (7.30-7.30) | < 0.001 |
| Serum bicarbonate, mEq/L | 23 (21-25) | 19 (16-21) | 23 (21-25) | < 0.001 |
| Serum electrolytes |  |  |  |  |
| Potassium, mmol/L | 4.8 (4.4-5.3) | 5.5 (5.0-6.4) | 4.8 (4.4-5.3) | < 0.001 |
| Sodium, mmol/L | 137 (135-139) | 136 ( 134-139) | 137 (135-139) | 0.008 |

Table 3. Logistic regression analysis with hospital mortality as the dependent variable in the whole population.

|  | **Univariate analysis** | | **Multivariable analysis** | |
| --- | --- | --- | --- | --- |
|  | **OR (95% CI)** | **p-value** | **OR (95% CI)** | **p-value** |
| Age (per year) | 1.03 (1.03-1.03) | < 0.001 | 1.04 (1.03-1.04) | < 0.001 |
| Male | 1.17 (1.04-1.31) | 0.009 | 1.03 (0.90-1.18) | 0.690 |
| SAPS II score (per point) | 1.06 (1.05-1.06) | < 0.001 | 1.05 (1.05-1.05) | < 0.001 |
| Comorbidities |  |  |  |  |
| Diabetes mellitus | 1.26 (1.12-1.41) | < 0.001 | 1.05 (0.92-1.21) | 0.481 |
| Arterial hypertension | 0.78 (0.67-0.90) | 0.001 | 0.99 (0.83-1.19) | 0.944 |
| Cirrhosis | 3.65 (2.96-4.50) | < 0.001 | 1.34 (1.01-1.77) | 0.042 |
| Cancer | 1.46 (1.28-1.67) | < 0.001 | 1.53 (1.29-1.81) | < 0.001 |
| Heart failure | 1.21 (1.03-1.42) | 0.020 | 1.37 (1.12-1.67) | 0.002 |
| Type of surgery |  |  |  |  |
| Digestive | R | NA | R | NA |
| Cardiothoracic | 0.58 (0.49-0.68) | < 0.001 | 0.28 (0.23-0.34) | < 0.001 |
| Neurosurgery | 0.22 (0.19-0.26) | < 0.001 | 0.84 (0.67-1.06) | 0.146 |
| Trauma | 0.24 (0.20-0.30) | < 0.001 | 0.89 (0.63-1.26) | 0.512 |
| SOFA subscores (per point) |  |  |  |  |
| Respiration | 1.29 (1.24-1.34) | < 0.001 | 1.06 (1.003-1.11) | 0.039 |
| Coagulation | 1.57 (1.48-1.67) | < 0.001 | 1.14 (1.05-1.23) | 0.001 |
| Liver | 1.86 (1.75-1.97) | < 0.001 | 1.35 (1.24-1.46) | < 0.001 |
| Cardiovascular | 1.57 (1.50-1.64) | < 0.001 | 1.37 (1.29-1.44) | < 0.001 |
| GCS | 1.29 (1.25-1.33) | < 0.001 | 1.23 (1.18-1.28) | < 0.001 |
| Emergency admissions | 1.88 (1.66-2.12) | <0.001 | 0.96 (0.83-1.12) | 0.633 |
| ESRD | 5.32 (3.92-7.24) | <0.001 | 3.84 (2.68-5.50) | < 0.001 |

OR: odds ratio; CI: confidence interval; ESRD: end-stage renal disease; SAPS II: Simplified Acute Physiology Score; SOFA: Sequential Organ Failure Assessment; GCS: Glasgow Coma Scale

Table 4. Summary of a multivariable forward stepwise logistic regression analysis* with in-hospital death as the dependent variable.

|  | **Odds ratio (95% CI)** | **p-value** |
| --- | --- | --- |
| Age, per year | 1.03 (0.99-1.06) | 0.137 |
| Female sex | 0.26 (0.10-0.66) | 0.005 |
| SAPS II score, per point | 1.02 (0.99-1.04) | 0.159 |
| SOFAhep subscore | 1.60 (1.07-2.40) | 0.022 |
| Diabetes mellitus | 0.43 (0.18-1.00) | 0.051 |
| Cardiac surgery | 0.28 (0.12-0.67) | 0.004 |
| Residual urine output |  |  |
| None | R | NA |
| < 500 mL/day | 0.59 (0.23-1.53) | 0.275 |
| > 500 mL/day | 0.16 (0.05-0.55) | 0.004 |
| Access for dialysis |  |  |
| A-V shunt | R | NA |
| Central venous catheter | 3.30 (1.36-8.04) | 0.009 |
| Peritoneal catheter | 4.23 (0.44-40.26) | 0.210 |

*Hosmer and Lemeshow, Chi Square 5.8; p-value = 0.67.

SAPS II: Simplified Acute Physiology Score; SOFA: Sequential Organ Failure Assessment.
